# An integrated somatic and germline approach to aid interpretation of germline variants of uncertain significance in cancer susceptibility genes

**DOI:** 10.3389/fonc.2022.942741

**Published:** 2022-08-25

**Authors:** Alison Schwartz, Danielle K. Manning, Diane R. Koeller, Anu Chittenden, Raymond A. Isidro, Connor P. Hayes, Feruza Abraamyan, Monica Devi Manam, Meaghan Dwan, Justine A. Barletta, Lynette M. Sholl, Matthew B. Yurgelun, Huma Q. Rana, Judy E. Garber, Arezou A. Ghazani

**Affiliations:** ^1^ Division of Cancer Genetics and Prevention, Dana-Farber Cancer Institute, Boston, MA, United States; ^2^ Department of Pathology, Brigham and Women’s Hospital, Boston, MA, United States; ^3^ Harvard Medical School, Boston, MA, United States; ^4^ Division of Genetics, Department of Medicine, Brigham and Women’s Hospital, Boston, MA, United States; ^5^ Division of Population Sciences, Dana-Farber Cancer Institute, Boston, MA, United States

**Keywords:** somatic and germline integration, tumor signature profile, germline VUS, lynch syndrome, paraganglioma, Li-Fraumeni syndrome

## Abstract

Genomic profiles of tumors are often unique and represent characteristic mutational signatures defined by DNA damage or DNA repair response processes. The tumor-derived somatic information has been widely used in therapeutic applications, but it is grossly underutilized in the assessment of germline genetic variants. Here, we present a comprehensive approach for evaluating the pathogenicity of germline variants in cancer using an integrated interpretation of somatic and germline genomic data. We have previously demonstrated the utility of this integrated approach in the reassessment of pathogenic germline variants in selected cancer patients with unexpected or non-syndromic phenotypes. The application of this approach is presented in the assessment of rare variants of uncertain significance (VUS) in Lynch-related colon cancer, hereditary paraganglioma-pheochromocytoma syndrome, and Li-Fraumeni syndrome. Using this integrated method, germline VUS in *PMS2*, *MSH6*, *SDHC*, *SHDA*, and *TP53* were assessed in 16 cancer patients after genetic evaluation. Comprehensive clinical criteria, somatic signature profiles, and tumor immunohistochemistry were used to re-classify VUS by upgrading or downgrading the variants to likely or unlikely actionable categories, respectively. Going forward, collation of such germline variants and creation of cross-institutional knowledgebase datasets that include integrated somatic and germline data will be crucial for the assessment of these variants in a larger cancer cohort.

## Introduction

The assessment of the pathogenicity of genetic variants associated with hereditary cancer predisposition is often limited due to the absence of key evidence in the literature, such as functional studies or the prevalence of disease alleles in familial cancer cohorts. In the clinical evaluation of germline alterations, the absence of these key evidence frequently renders the classification of variants of uncertain significance (VUS). Indeed, VUS constitutes a significantly large proportion of reported variants in large-scale genome studies (1, 2), and accounts for approximately 50% of variants in ClinVar (3). VUS designation is not informative or actionable in the clinical management of genetic disease, and carries a high clinical and emotional burden (4, 5). Therefore, there is an active need to systematically reclassify VUS to actionable or non-actionable categories in all genetic conditions.

Among patients affected by hereditary genetic conditions, cancer patients present a unique population, as they have two sets of informative genomes: somatic and germline. While the application of joint analysis of somatic and germline data has been demonstrated in personalized therapeutic practice (1, 6–13), tumorigenesis and cancer progression (14–20), inference on germline allele penetrance (21–23) and gene or biomarker discovery (24–26), it remains underutilized in clinical practice (27) and in the assessment of pathogenicity of germline variants. Signature somatic profiles such as loss of heterozygosity (LOH), mismatch repair-deficient (MMRd) or mismatch repair-proficient (MMRp) status, microsatellite instability (MSI), and tumor-derived information such as immunohistochemistry (IHC) of target proteins have unique values in specific cancer types that can shed light on the role of germline genetic variants in disease.

We have previously demonstrated the value of tumor-derived somatic data in assessing germline variants in patients who do not exhibit classic syndromic phenotypes for clinical diagnosis of von Hippel-Lindau syndrome and APC-associated colon cancer (21–23, 28). Taking advantage of signature tumor profiles, here we present a cancer-specific approach in assessing tumor-derived data as supporting evidence in the assessment of pathogenicity of germline VUS. Specifically, we focused on variants in genes that are associated with three hereditary cancer syndromes: Lynch syndrome (LS), paraganglioma-pheochromocytoma (PGL/PCC) syndromes, and Li-Fraumeni syndrome (LFS). Using unique tumor signature data, molecular pathology, mechanism of tumorigenicity, and patient clinical presentation, we demonstrate a comprehensive method for reassessing VUS in these specific cancer types. The goal is to utilize the collection of this evidence to inform the function of VUS and classify them as variants that are either considered presumed deleterious (upgrading the classification) or non-deleterious (downgrading the classification).

## Results

### Patient cohort

**Figure 1:**
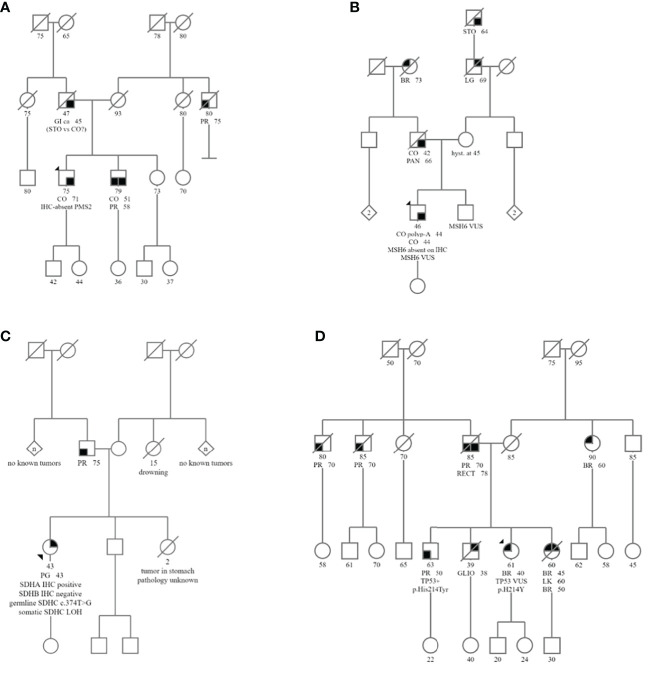
Pedigrees of Subjects 1-4 are shown in **(A–D)**, respectively. Abbreviated cancer types: CO, Colon cancer; BR, Breast cancer; PR, Prostate cancer; PG, Paraganglioma; GI, GI cancer unspecified; STO, Stomach cancer; CO polyp- A, Adenomatous colon polyp; RECT, Rectal cancer; PAN, Pancreatic cancer; LG, Lung cancer; LK, Leukemia; GLIO, Glioblastoma.

The study cohort consisted of patients who presented at the Division of Cancer Genetics and Prevention, Dana-Farber Cancer Institute (DFCI) for genetic evaluation, and at the Pathology Department, Brigham and Women’s Hospital (BWH) for tumor evaluation between 2016 to 2021. We sought to evaluate individuals with rare germline VUS in MMR genes (*PMS2*, *MLH1*, *MSH2*, or *MSH6*), *SDHx* genes *(SDHA, SDHB, SDHC, SDHD, or SDHAF2)*, and *TP53* gene in individuals who are suspected of having LS, hereditary PGL/PCC, and LFS, respectively. The availability of full tumor and somatic genetic data was a requirement in our approach. Based on this genetic evaluation, four subjects (Subjects 1-4) were identified to carry germline VUS of high concern (**Table 1**; **Figure 1**).

**Table 1:** Study subjects clinical characteristics and cancer history.

Subjects	Age at dx (current age )	Sex	Tumor type/detail	Related syndromic tumor/phenotype in family
Subject 1	71 (75)	M	Colonic Adenocarcinoma; Right Colon; Low Grade	Brother- colon cancer age at 53; Father- stomach/colon cancer in 40s
Subject 2	44 (46)	M	Colonic Adenocarcinoma	Father-colon cancer at 42, pancreatic cancer at 66
Subject 3	43 (49)	F	Right Atrial Paraganglioma	Not available
Subject 4	40 (61)	F	Invasive Ductal Carcinoma - Right Breast; Colorectal Carcinoma	Brother- glioblastoma at 38, positive for TP53:c.640C>T; Sister- breast cancer at 45 and contralateral breast cancer at 50, and leukemia at age 60
Subject 5	59 (61)	M	Intrahepatic Cholangiocarcinoma	None
Subject 6	69 (71)	M	Lung Adenocarcinoma	Father-liver cancer at unknown age
Subject 7	49 (53)	M	Metastatic Clear Cell Renal Cell Carcinoma	Brother-prostate cancer at unknown age
Subject 8	53 (58)	M	Metastatic Clear Cell Renal Cell Carcinoma (to lung, femur, and maxilla)	No related cancer known
Subject 9	62 (66)	F	Endometrial Adenocarcinoma, Serous Type	Paternal aunt-abdominal or liver cancer, d.85; Paternal aunt-abdominal or liver cancer, d.85; Maternal first cousin-liver cancer, d.56
Subject 10	59 (61)	M	Metastatic Poorly Differentiated Carcinoma, Urothelial Origin	No related cancers
Subject 11	54 56)	F	Diffuse Type Stomach Adenocarcinoma	No family history of SDH-associated tumors
Subject 12	63 (66)	F	Leiomyosarcoma	No family history of SDH-associated tumors
Subject 13	63 (66)	F	Uterine Adenosarcoma	No family history of SDH-associated tumors
Subject 14	69 (73)	F	Urothelial Carcinoma	Sister with colon cancer at unknown age. No documented family history of Li-Fraumeni syndrome core tumors
Subject 15	50 (53)	F	Angiosarcoma	Brother with melanoma at age 54. No documented family history of Li-Fraumeni syndrome core tumors
Subject 16	56 (58)	F	Intrahepatic Cholangiocarcinoma	Colon cancer in paternal aunt unknown age; Melanoma in sister unknown age. No documented family history of Li-Fraumeni syndrome core tumors

To expand our search, we then performed a systematic query of the PROACTIVE (Profile And Cancer gene Testing for IndiVidual Evaluation) database of germline VUS in MMR, *SDHx*, and *TP53* genes in any individual regardless of cancer types. The PROACTIVE study is a DFCI institute-wide research study that collects germline genetic data from patients with cancers of diverse types, with a focus on patients considered low-risk for established hereditary cancer predisposition syndromes. The PROACTIVE query captured subjects whose germline and somatic testing was performed at the Center for Advanced Molecular Diagnostics (CAMD) at BWH between 2019 and 2021. Twelve subjects were identified with VUS in the above-noted genes but with cancers unrelated to these genes (Subjects 5-16). A comprehensive list of study Subjects is shown in **Table 1**.

### Clinical characteristics of study subjects

**Figure 2:**
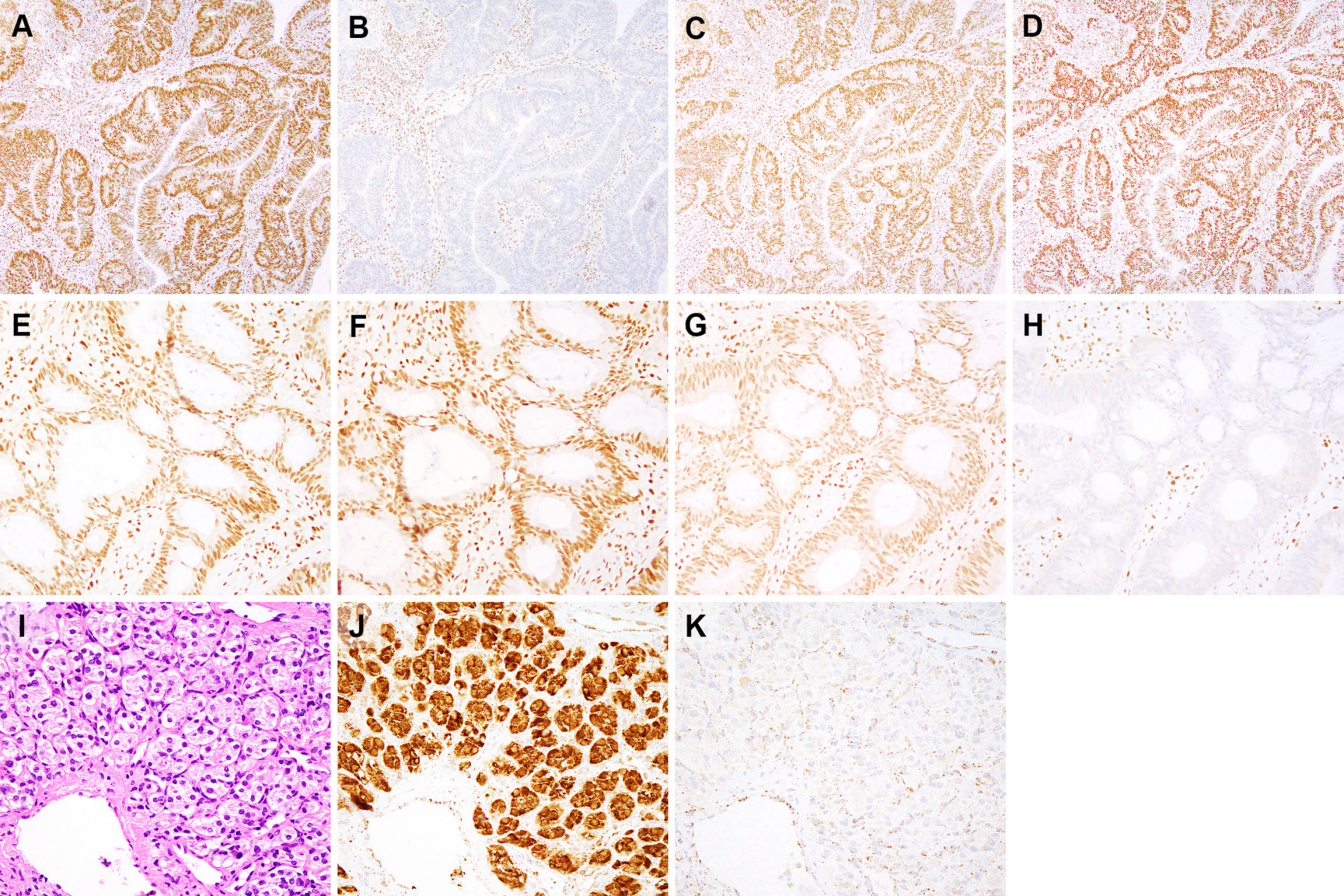
Immunohistochemical findings for subjects 1-3. Immunohistochemistry for mismatch repair proteins (MMR) performed on the colonic adenocarcinoma from subject 1 **(A–D)** demonstrates intact nuclear staining for MLH1 **(A)**, MSH2 **(C)**, and MSH6 **(D)** in both tumor and stromal cells. Staining for PMS2 **(B)** is lost in tumor cells and retained in stromal cells. MMR immunohistochemistry performed on the colonic adenocarcinoma from subject 2 **(E–H)** shows loss of MSH6 expression in tumor cell nuclei and retained expression in stromal cell nuclei **(H)**. MLH1 **(E)**, PMS2 **(F)** and MSH2 **(G)** show retained nuclear expression in both tumor and stromal cell nuclei. This isolated loss of PMS2 **(B)** and MSH6 **(H)** in tumor cells, as demonstrated by immunohistochemical staining, is typically seen in cases with *PMS2* and *MSH6* germline mutations, respectively (29). Only tumor/neoplastic cells show loss of staining as they contain an inherited mutant allele (first hit) and an allele that is inactivated during tumorigenesis (second hit). The paraganglioma from subject 3 **(I)**, hematoxylin & eosin) shows nests of cuboidal cells with associated blood vessels. Immunohistochemical staining for SDHA **(J)** shows strong staining in tumor and endothelial cells, whereas SDHB staining is lost in tumor cells and expressed in stromal and endothelial cells **(K)**. Immunohistochemical expression of SDHB is lost whenever there is biallelic inactivation of any component of the *SDHx* complex, while SDHA expression is lost when *SDHA* undergoes biallelic inactivation (30). **(A–D)**, 100x magnification. **(E–H)**, 200x magnification. **(I–K)**, 400x magnification.

Subject 1 was diagnosed at age 71 with MMRd colon adenocarcinoma with loss of PMS2 staining by IHC (**Table 2**; **Figures 2A–D**). Germline genetic testing revealed a VUS, *PMS2*:c.716T>G. Subject 1 met the Amsterdam II criteria for LS based on his personal history of colon adenocarcinoma, and two first-degree relatives in two successive generations with LS-associated cancers with one relative diagnosed before age 50 (**Figure 1**). Somatic OncoPanel test identified the above germline *PMS2*:c.716T>G variant, due to the presence of germline cells in the tumor biopsy. Somatic OncoPanel also detected a somatic *PMS2*:c.904-2A>C variant. Full clinical history for Subject 1 is available in **Supplementary Table 1**, and variant annotation data for germline VUS are shown in **Supplementary Table 2**.

**Table 2:** Comprehensive tumor-derived somatic and germline data of study subjects.

Subjects	Cancer history (phenotypic assessment for cancer predisposition	Germline alteration (top)	MMR/MS status	IHC in relevant tumor tissue	Comment
		Allelic Somatic alteration(s) in tumor tissue (bottom)			
**Subject 1**	Colon cancer (meets Amsterdam criteria)	*PMS2*: c.716T>G (p.Leu239Arg)	MMR-D/ MSI-H	Colon biopsy: Loss of PMS2; Intact MLH1, MSH2, and MSH6	PMS2 deficiency by IHC is consistent with biallelic *PMS2* inactivation; tumor is negative for *BRAF:c.1799T>A* Val600Glu; IHC, somatic signature and MMR status are consistent with Lynch syndrome
		Colon biopsy: *PMS2*:c.904-2A>C			
**Subject 2**	Colon cancer (Bethesda criteria; PREMM5 score 29.2%)	*MSH6*:c.1439_1441dup (p.Val480dup)	MMR-D/ MSI-H	Colon biopsy: Loss of MSH6; Intact MLH1, MSH2, and PMS2	MSH6 deficiency by IHC is consistent with biallelic *MSH6* inactivation; tumor is negative for *BRAF:c.1799T>A* Val600Glu; IHC, somatic signature and MMR status are consistent with Lynch syndrome
		Colon biopsy: *MSH6*:c.3253_3254insC (p.Phe1088Leufs*5); *MSH6* c.3556+2T>C; *MSH6*:c.3173A>T (p.Asp1058Val)			
**Subject 3**	Paraganglioma (tumor type suggestive of hereditary PPGL)	*SDHC*: c.374T>G (p.Met125Arg)	Not applicable	Myocardial valvular biopsy: SDH-deficient	SDHB deficiency by IHC is consistent with biallelic *SDHC* inactivation
		Myocardial valvular biopsy: Monosomy of chromosome 1 that includes one copy loss of *SDHC at* 1q23.3			
**Subject 4**	Breast cancer (meets Chompret criteria for LFS)	*TP53*:c.640C>T (p.His214Tyr)	MMR-P/ MSS	Breast biopsy: HER2/neu (c-erb-B2) 3+; Colon biopsy: intact nuclear staining for MLH1, MSH2, MSH6, and PMS2	Strong proband and family phenotypic presentations, and biallelic inactivation of *TP53* are consisted with Li-Fraumeni syndrome. MMR-P/MSS and intact IHC rule out Lynch-related colon cancer
		Colon biopsy: one copy deletion of *TP53* at 17p13.1			
**Subject 5**	Cholangiocarcinoma (No criteria met)	*PMS2*:c.620G>A (p.Gly207Glu)	MMR-P/ MSS	Liver core biopsy: Positive - CK20(weak); Negative - CK7, CDX-2, TTF1	Somatic alteration possibly consistent with sporadic cholangiocarcinoma: *BRAF* fusion (*BRAF* intron 9 :: *ZBTB16* intron 3)
		None			
**Subject 6**	Prostate cancer (No criteria met)	*PMS2*:c.1688_1689delinsAG (p.Arg563Gln)	MMR-P/ MSS	Lung core biopsy: Positive: TTF-1, Napsin A; Negative: PAX8, thyroglobulin	Somatic alteration consistent with sporadic lung cancer: oncogenic *EGFR* c.2235_2255delinsAAT (p.Glu746_Ser752delinsI); deletion - *EGFR* exon 19
		None			
**Subject 7**	Malignant seminoma (No criteria met)	*MSH6*:c.1028C>T (p.Pro343Leu)	MMR-P/ MSS	Renal mass biopsy: Positive - PAX8, CA9; Negative - SALL4, OCT3/4, PU.1, AE1/AE3, Inhibin, S100, HMB-45, TFE3	Somatic alteration consistent with sporadic *VHL*-negative RCC tumors: *NF2*:c.1259del (p.Glu420Glyfs*6)
		None			
**Subject 8**	RCC (No criteria met)	*MSH6*:c.494T>G (p.Phe165Cys)	Unavailable	Pulmonary wedge resection: Positive - PAX8; Negative - TTF-1	Somatic alterations consistent with sporadic RCC*: VHL*:c.266T>A (p.Leu89His); *PBRM1*:c.292dupC (p.Gln98Profs*10); low level gain of a portion of chromosome 5q; one copy loss of chromosome 3p (including *VHL, SETD2*, and *PBRM1*)
		None			
**Subject 9**	Endometrial carcinoma (PREMM5 score 2.8%)	*MSH6*:c.1774G>A (p.Val592Ile)	MMR-P/ MSS	Endometrium biopsy: intact MLH1, MSH2, MSH6, and PMS2; Uterine biopsy: HER2/NEU Positive (3+)	MMR-P/MSS, intact IHC, and absence of biallelic inactivating of MSH6 rule out lynch-related endometrial cancer. Somatic alterations consistent with sporadic serous-like endometrial carcinoma and uterine carcinoma: *PIK3CA*:c.1635G>T (p.Glu545Asp); *TP53*:c.817C>T (p.Arg273Cys); gain of 3q26.2; Low copy number gain of *CCNE1* at 19q12
		None			
**Subject 10**	Urothelial carcinoma (No criteria met)	*MSH6*:c.3256C>T (p.Pro1086Ser)	MMR-P/ MSS	Liver biopsy: Positive - GATA3, CK7 (multifocal); Negative - CK20, PAX8, TTF1, CDX2	Somatic alterations consistent with sporadic urothelial carcinoma: *KRAS*:c.35G>A (p.Gly12Asp); *TP53*:c.725G>A (p.Cys242Tyr); Two-copies deletion of *CDKN2A* at 9p21.3; single copy deletion of *TP53* at 17p13.1
		None			
**Subject 11**	Gastric adenocarcinoma (No personal history of PPGL)	*SDHA*:c.1579C>T (p.Arg527Cys)	MMR-P/ MSS	Antral mass biopsy: Positive - AE1/AE3	Somatic alterations consistent with sporadic gastric adenocarcinoma: high copy number gain of *GATA4* at 8p23.1; high copy number gain of *NEIL2* at 8p23.1
		None			
**Subject 12**	Retroperitoneal leiomyosarcoma diagnosed at 63 (no PPGL).	*SDHC*:c.430G>C (p.Glu144Gln)	MMR-P/ MSS	Mesenteric mass biopsy: Positive - Caldesmon, SMM; Negative - ER, PR; Negative - DOG-1, KIT, PDGFR, SDHB (retained)	Somatic alterations consistent with sporadic leiomyosarcoma: *TP53*:c.794T>G (p.Leu265Arg); single copy deletion of *RB1* at 13q14.2; 153bp deletion involving intron 19 of *RB1*
		None			
**Subject 13**	Uterine adenosarcoma diagnosed at age 63. No personal history of PPGL?SDH-associated tumors .	*SDHC*:c.430G>C (p.Glu144Gln)	MMR-P/ MSS	Endometrium biopsy: Positive - Desmin; Negative - AE1/AE3, CD117, CD10, cyclinD1, EMA, ER, PR, SMA, MART1, S100; Vimentin is non-contributory	Somatic alteration not consistent with biallelic SDHC inactivation: nonspecific regional low-level gains and focal low-level losses across the targeted genome
		Low amplification of 1q23.3 region including *SDHC* (non-specific)			
**Subject 14**	Urothelial Carcinoma diagnosed age 69; Carcinoid tumor of lung age 70; breast cancer triple negative age 56. No LFS core tumors (except for breast cancer, however diagnosed at later age than expected with LFS).	TP53:c.-29+236T>C TP53:c.-29+571T>C	MMR-P/ MSS	Urethral biopsy: Positive - PAX8; Negative - TTF-1, GATA3	Somatic alterations consistent with sporadic urothelial carcinoma: moderate copy number gain of *PRDM1* at 6q21: *PRDM1*; Single copy deletion of *TP53* at 17p13.1; tumor is likely sporadic, no germline driven LOH
		Single copy deletion of *TP53* at 17p13.1			
**Subject 15**	No LFS core tumor. Angiosarcoma, not typical sarcoma types in LFS TP53 P/LP carriers	*TP53*:c.-29+1044T>A	MMR-P/ MSS	Diaphragm tumor resection: Positive - ERG, CD31	Somatic alterations consistent with sporadic angiosarcoma: oncogenic *NRAS*:c.181C>A (p.Gln61Lys); *TP53*:c.404G>A (p.Cys135Tyr); two-copy deletion of *CDKN2A* and *CDKN2B* at 9p21.3; two-copy deletion of *TSC2* at 16p13.3; single copy deletion of *TP53* at 17p13.1; tumor is likely sporadic, no germline driven LOH
		*TP53*:c.404G>A (p.Cys135Tyr); Single copy deletion of *TP53* at 17p13.1			
**Subject 16**	No LFS core tumors	*TP53*:c.-29+1044T>A	MMR-P/ MSS	Liver core biopsy: Positive - CK7, SMAD4 (loss); Negative - CK20, CDX2, TTF-1, PAX8	Somatic alterations consistent with sporadic cholangiocarcinoma: oncogenic KRAS c.35G>A (p.Gly12Asp); *TP53*:c.329G>T (p.Arg110Leu); Single copy deletion of *ARID1A* at 1p36.11; Single copy deletion of *TP53* at 17p13.1; Tumor is likely sporadic, no germline driven LOH
		*TP53*:c.329G>T (p.Arg110Leu); Single copy deletion of *TP53* at 17p13.1			

IHC, Immunohistochemistry; LFS, Li-Fraumeni syndrome; LOH, Loss of heterozygosity; MMR, Mismatch repair; MMR-D, Mismatch repair-deficient; MMR-P, Mismatch repair-proficient; MS, Microsatellite stability; MSI-H, Microsatellite instable-high; MSS, Microsatellite stable.

Subject 2 was diagnosed at age 44 with MMRd colon adenocarcinoma with loss of MSH6 staining by IHC (**Table 2**, **Figures 2E–H**). Germline genetic testing identified a VUS, *MSH6:*c.1439_1441dup. Somatic OncoPanel identified the following somatic variants in *MSH6*: c.3253_3254insC (p.F1088Lfs*5); c.3556+2T>C; and c.3173A>T (p.D1058V) The Revised Bethesda criteria were met for this patient based on his diagnosis of colorectal cancer under age 50. Additionally, a first-degree relative was diagnosed with two LS-associated tumors, with colon cancer at age 42 and pancreatic cancer at age 66 (**Figure 1**). Based on the personal and family history of subject 2, the likelihood of a LS mutation was 29.2% by the PREMM5 model. Full clinical history for Subject 2 is available in **Supplementary Table 1**, and variant annotation data for germline VUS are shown in **Supplementary Table 2**.

**Figure 3:**
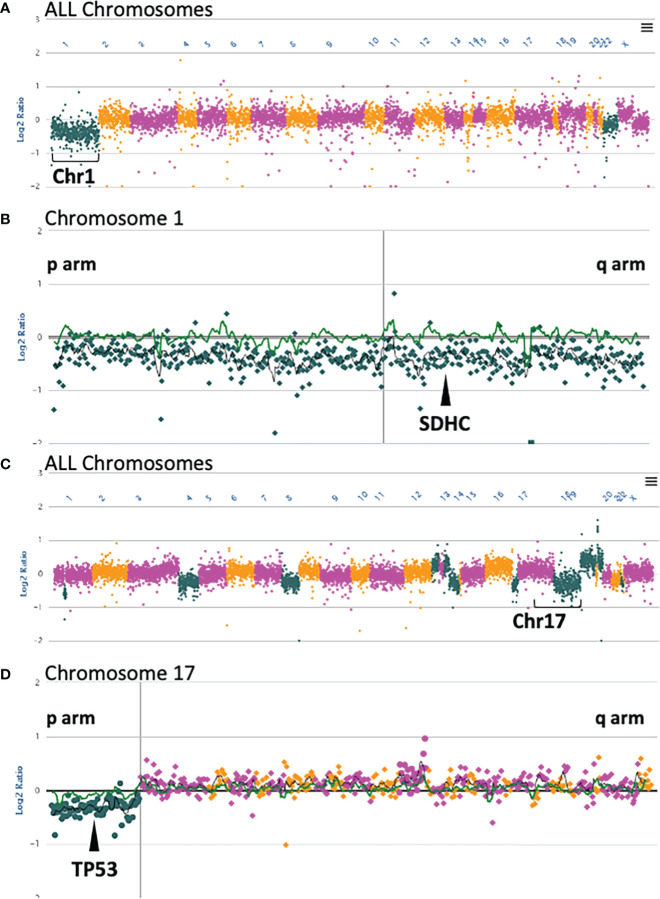
Somatic OncoPanel copy number alterations in Subject 3 and Subject 4. **(A)** All chromosome view of copy number analysis of the Subject 3 somatic sample showing single copy loss of Chromosome 1. **(B)** Single copy loss of Chromosome 1 encompasses *SDHC* at position 1q23.3. **(C)** All chromosome view of copy number analysis of the Subject 4 somatic sample showing several gains and losses, including loss of Chromosome 17p. **(D)** Single copy loss of Chromosome 17p includes *TP53* at position 17p13.1. Each dot represents a contiguously baited segment. The read counts of each segment were normalized against a panel of normal samples to plot the Log2 ratios. The positions of the genes are relative to the targeted loci in the panel. The vertical lines in **(B, D)** represent the centromere in each chromosome. Copy number plots were manually reviewed, and calls were made with an adaptive calling method that adjusts the threshold per sample.

Subject 3 was diagnosed at age 43 with a right atrial paraganglioma. IHC showed intact staining for SDHA and absent staining for SDHB (**Table 2**; **Figure 2**). Germline genetic testing revealed a VUS, *SDHC:*c.374T>G. Somatic OncoPanel showed a one copy loss of *SDHC* (**Figure 3**). No related syndromic tumor or phenotype in the family was reported (**Table 2**; **Figure 1**). Full clinical history for Subject 3 is available in **Supplementary Table 1**, and variant annotation data for germline VUS are shown in **Supplementary Table 2**.

Subject 4 was diagnosed at age 40 with ER-positive, PR-positive, and HER2/neu-positive invasive ductal carcinoma of the breast (**Table 2**). Genetic testing revealed a VUS, *TP53*:c.640C>T, that was confirmed to be germline by positive family member testing. The Chompret (2015) criteria for LFS were met with a personal history of breast cancer diagnosed before age 46 and a first-degree relative with an LFS component tumor before age 56 which includes a brother with glioblastoma at age 38 (**Figure 1**). Additional family history of cancer within the LFS tumor spectrum includes a sister with two separate breast malignancies at ages 45 and 50, and acute myeloid leukemia at age 60. Subject 4 was recently diagnosed with MMR proficient colon adenocarcinoma at age 60 following colonoscopy and EGD screening due to the germline *TP53* result with clinical concern for LFS. Somatic OncoPanel on colon biopsy showed a one copy number loss in *TP53* (**Figure 3**). Full clinical history for Subject 4 is available in **Supplementary Table 1**, and variant annotation data for germline VUS are shown in **Supplementary Table 2** and in **Supplementary Table 3**.

### Reassessment of germline variants

The following section addresses the re-assessment of germline variants of uncertain significance using our approach, based on the clinical data, tumor type, personal and family history, and tumor signature profiles, and IHC patterns.

#### Assessment of germline variants of uncertain significance in MMR genes in patients with LS-associated colon cancer

The mechanism of tumorigenicity of Lynch-related colon cancer is well known to involve mismatch repair (MMR) genes. Selected tumor tests are routinely used to assess and diagnose LS: MMR status, MSI, biallelic inactivation of MMR genes, tumor signature, and IHC staining of MMR gene expression (31, 32). To re-assess the classification of germline VUS in MMR genes, we leveraged the collection of tumor-derived information along with the patients’ personal and family history information. Germline testing revealed *PMS2*: c.716T>G in Subject 1 and *MSH6*:c.1439_1441dup in Subject 2 (**Table 2**). Due to the absence of ample ACMG-based evidence, the variants were classified as VUS by the reporting laboratories (**Supplementary Table 3**). Subjects 1 and 2 presented with colon adenocarcinoma and a strong family history of LS-associated cancers (Amsterdam II and Bethesda criteria in Subject 1 and Subject 2, respectively).

Somatic OncoPanel testing on DNA extracted from colon cancer biopsy identified somatic *PMS2*:c.904-2A>C in Subject 1. The *PMS2*:c.904-2A>C is an inactivating variant in the canonical splice site in exon 9 of 15, expected to cause aberrant splicing, resulting in the loss of protein product from that allele due to nonsense-mediated mRNA decay (**Supplementary Table 3**). OncoPanel was negative for *BRAF:c.1799T>A* Val600Glu (**Table 2**), an observation that provides evidence against colon cancer of somatic origin.

In subject 2, OncoPanel testing on DNA extracted from colon cancer biopsy identified three somatic variants in *MSH6*: *MSH6*:c.3253_3254insC, *MSH6*:c.3556+2T>C, and *MSH6*:c.3173A>T (**Table 2**; **Supplementary Table 3**). The MSH6:c.3253_3254insC is frameshift variant resulting in a premature stop codon five residues downstream (p.F1088Lfs*5). Although this is an inactivating variant, it is located at a homopolymer site, and it mostly likely reflects a passenger event due to MSI. The somatic *MSH6*:c.3173A>T variant is likely deleterious. The somatic splice site *MSH6*:c.3556+2T>C variant is an inactivating variant in the canonical splice site in exon 5 of 10 that is expected to produce a transcript subjected to nonsense-mediated mRNA decay. This variant, therefore, likely represents the critical second hit. The tumor biopsy was also negative for *BRAF* Val600Glu (**Table 2**), an observation that rules out somatic colon cancer.

Both tumors presented MMR deficient and microsatellite instable features (i.e., MMR-D/MSI-H). The IHC staining of the colon biopsy showed deficiency for PMS2 (Subject 1) and MSH6 (Subject 2) proteins, with intact status for other MMR proteins (**Figures 2A–H**), a pattern consistent with LS-associated colon cancer with germline involvement as a first hit (29). The collective sum of these tumor-derived somatic data provided evidence that these germline variants are likely inactivating variants serving as the first hit in the “two-hit” model of tumorigenicity in the given MMR genes. Therefore, the germline variants in the *PMS2* (in Subject 1) and *MSH6* (in Subject 2) genes, previously classified as VUS, were reclassified to presumed deleterious and therefore considered actionable (**Table 2**). Based on these findings and in the context of the personal and family history of colon cancer, these patients have been managed with a presumed diagnosis of LS.

#### Assessment of germline variants of uncertain significance in MMR genes in patients with non-LS associated tumors

The query of the DFCI database did not show any additional individuals positive for the *PMS2*: c.716T>G and *MSH6*:c.1439_1441dup variants. We further queried the PROACTIVE (21–23) database for any germline VUS in any of the MMR genes. The query revealed six Subjects (Subject 5 - Subject 10) positive for germline VUS in *PMS2* or *MSH6* genes who had non-Lynch related tumors of cholangiocarcinoma, lung adenocarcinoma, clear cell renal cell carcinoma, serous type endometrial adenocarcinoma, and urothelial carcinoma (**Tables 1**, **2**). A list of germline variants for the Subjects is shown in **Supplementary Table 3**.

Detailed evaluation of clinical data on each Subject showed a personal and family history profile inconsistent with LS, absence of biallelic involvement of MMR genes, and tumor profiles consistent with proficient MMR and microsatellite stable tumor (MMR-P/MSS). Altogether, there was no clinical or genomic evidence indicating the involvement of LS. In fact, the examination of somatic data showed key signature profiles consistent with sporadic tumors in each patient (**Table 2**). There was no evidence of germline contribution to LS in any Subjects, and therefore, the germline VUS in these patients was unlikely to be deleterious. Using this approach, we could present supporting evidence for downgrading the classification of these VUS in *PMS2* or *MSH6* genes.

#### Assessment of germline variant of uncertain significance in succinate dehydrogenase x (*SDHx*) genes in patient with PGL/PCC associated cancer

The mechanism of tumorigenicity in SDH-associated hereditary PGL/PCC syndromes includes the inactivation of *SDHx* genes. The unique feature of SDH-related PGL/PCC is the function of subunits of the succinate dehydrogenase (SDH) in mitochondrial respiratory chain complex II. If any component of the SHD subunit is completely inactivated, the entire complex becomes unstable, resulting in the degradation and loss of the SDHB subunit. SDHB staining has high sensitivity and specificity for the presence of any SDH mutation (33). Therefore, SDHB-absent tumor immunostaining suggests the presence of an inactivating germline *SDHA, SDHB, SDHC*, or *SDHD* followed by a somatic loss in the second allele of the gene (30, 33–35).

We used this signature feature of PGL/PCC, and detailed patients’ personal and family history, to assess a germline VUS in the *SDHC* gene (Subject 3, **Table 2**). Subject 3 had an early diagnosis of atrial paraganglioma, a rare primary cardiac tumor (36, 37). Genetic testing revealed a germline *SDHC*:c.374T>G and somatic monosomy of chromosome 1 (**Figure 3**). The one copy deletion of chromosome 1 results in one copy loss of *SDHC* gene, as *SDHC* resides at 1q23.3. The SDHB-deficient IHC on the myocardial valvular biopsy (**Figure 2I–K**) confirmed that the germline variant is likely an inactivating first hit in the *SHDC* gene, and is likely involved in this patient’s paraganglioma. Based on the totality of this evidence, the germline *SDHC:*c.374T>G variant, previously classified as VUS by the clinical laboratory, was classified as presumed deleterious and treated as a clinically significant finding in relation to hereditary paraganglioma in this patient.

#### Assessment of germline variant of uncertain significance in succinate dehydrogenase x (*SDHx*) genes in patient with non-PGL/PCC associated cancer

The query in the DFCI database did not show additional individuals with *SDHC*:c.374T>G. A broader search in the PROACTIVE database was performed for VUS in any SDH*x* genes. Three Subjects with VUS were identified: *SDHA*: *SDHA* c.1579C>T in Subject 11, or *SDHC*:c.430G>C in Subjects 12 and 13, with respectively the diagnosis of gastric adenocarcinoma, retroperitoneal leiomyosarcoma, and uterine adenosarcoma (**Table 2**). A list of germline variants for the Subjects is shown in **Supplementary Table 3**. No personal or family history data supported the involvement of PGL/PCC syndromes in these patients (**Table 1**). There was no evidence of allelic involvement in tumor biopsies. Moreover, the tumor genomic profile was consistent with sporadic gastric adenocarcinoma (in Subject 11), and sporadic leiomyosarcoma (in Subject 12). The somatic alterations were nonspecific in uterine adenosarcoma (Subject 13). The totality of this evidence suggested that germline variants *SDHA*:c.1579C>T (p.Arg527Cys) and *SDHC*:c.430G>C are unlikely to be involved in PGL/PCC in these patients, supporting downgrading the classification of these VUS in hereditary PGL/PCC (**Table 2**).

#### Assessment of germline variant of uncertain significance in TP53 gene in patients suspected of LFS

The mechanism of tumorigenicity in LFS is the biallelic inactivation of TP53. Given LFS is associated with many different tumor types, and given that somatic TP53 alterations are very common in tumors, our approach for the assessment of germline VUS in *TP53* in the context of LFS required the presence of personal and family history suggestive of LFS. Subject 4 was positive for germline *TP53*:c.640C>T, classified as VUS by the reporting laboratory. The patient had a personal history of breast cancer diagnosis at age 40, a brother with glioblastoma at age 38, a sister with breast cancer at ages 45 and contralateral breast cancer at age 50, and leukemia at age 60 (**Figure 1**; **Table 1**).

Based on this personal and family history, Subject 4 met the Chompret criteria for LFS. The collection of these clinical data suggested the germline *TP53*:c.640C>T is likely a deleterious variant in this patient, involved in LFS (**Table 2**).This prompted a follow-up for the patient with LFS management guidelines including a colonoscopy which revealed a stage IIIb adenocarcinoma of the ascending colon. Genetic evaluation on colon biopsy showed a one copy deletion in 17p13.1 that included *TP53* (**Figure 3**); tumor profile was MMRp/MSS. Nuclear staining of colon biopsy showed intact MMR staining for mismatch repair proteins of *MLH1, MSH2, MSH6*, and *PMS2*, which, combined with the MSS profile on sequencing, ruled out Lynch-related colon cancer (**Table 2**).

#### Assessment of germline variant of uncertain significance in TP53 gene in patients not suspected of LFS

The query in the DFCI database did not show additional individuals with germline *TP53*:c.640C>T. A broader search in the PROACTIVE database identified three individuals (Subjects 14 - Subjects 16) positive for another germline VUS in *TP53* (**Table 2**). The Subjects respectively presented with urothelial carcinoma, angiosarcoma, and cholangiocarcinoma (**Table 1**). None of these cancers are among tumors associated with LFS. There was no documented family history of LFS core tumors. A list of germline variants for the Subjects, in addition to the VUS, is shown in **Supplementary Table 3**. All tumor biopsies were positive for somatic *TP53* single nucleotide variants (SNVs) (Subject 14) and one copy number loss of *TP53* (Subjects 15 and Subjects 16). Somatic alterations in *TP53* in tumors are very common. Therefore, the presence of these *TP53* alterations alone was not considered as supporting evidence for germline *TP53* involvement as the first hit. Tumor genomic profiling showed signature alterations consistent with sporadic urothelial carcinoma (Subjects 14), sporadic angiosarcoma, (Subjects 15), and sporadic cholangiocarcinoma (Subjects 16), the collective sum of evidence suggests these germline *TP53* variants are not involved in LFS tumorigenicity, and they are unlikely to be actionable (**Table 2**).

## Discussion

The ACMG/AMP guideline (38) was developed to evaluate the pathogenicity of germline sequence variants in Mendelian disease. However, in cancer, the tumor genome has a wealth of information about cancer progression that does not necessarily fit in the rule-based infrastructure developed for germline variant interpretation in hereditary conditions. Indeed, the full extent of the consequence of germline findings can often be appreciated when examined with somatic genetic data in unison. Several approaches have been reported to manage germline findings in cancer by integrated analyses of germline and somatic data (39–44). Some of these strategies use a one-for-all approach in cancers (39, 40) or assign ACMG criteria - developed for germline disease - to somatic data (e.g., somatic hotspots) (39, 42). Currently, a systematic evidence-based approach for a joint interpretation of germline and somatic data does not exist. Both tumor-derived genomic signatures and pathology examination of tumor biopsies are unique to cancer types (45) or cancer subtypes (41). The tumor-derived somatic evidence has a variable degree of relevance in different cancers, and one generalized approach of variant assessment is not practical for all cancer types. Therefore, we took a cancer-specific approach in our integrated germline variant interpretation. We captured the uniqueness of each tumor type, tumor signature profile, known mechanisms of tumorigenicity, clinical presentation of cancer, and we used the collection of this information to inform the role of germline VUS in disease.

The strength of our approach presented here is that it utilizes empirical evidence, well-established tumor molecular features (e.g., MMR, MSI, LOH) and histopathological results, combined with well-defined hereditary cancer syndrome clinical criteria (e.g., Amsterdam Criteria, Revised Bethesda Criteria). In the aggregate, positive findings are pathognomonic for inherited disease and serve as strong evidence supporting the pathogenicity of germline variants. We have previously demonstrated that this comprehensive approach differentiates between non-syndromic sporadic tumors or syndromic cancer (21, 22, 28). Examples include sporadic clear cell renal cell carcinomas with somatic-only biallelic inactivation of *VHL* (46–48) or sporadic hemangioblastomas with or without the presence of LOH in the *VHL* gene (49), and not related to syndromic VHL disease. In the assessment of the prevalence of a genetic variant in related individuals (in reporting segregation studies), or unrelated individuals (in reporting case-control studies), it is pivotal to determine if the evaluation is done in the context of a syndromic disease (e.g., VHL syndrome), or sporadic tumors. The former may suggest constitutional genetic involvement, whereas the latter, if used mistakenly, may lead to an over-classification of pathogenicity of the variants.

It is noteworthy that this integrated somatic and germline approach here requires an expert investigation of the full spectrum of somatic genomic signatures. As an example, while MSI is a well-known marker of LS-related tumors, it is not unique to LS, as 80% of MSI/mismatch repair-deficient (MMRd) tumors are sporadic (50). However, the combined presence of MMR/MSI, LOH and IHC and positive clinical criteria for LS in the patient is highly indicative of germline involvement. Conversely, selective tumor markers are only present in sporadic tumors and can be used as exculpatory evidence to rule out the hereditary involvement in cancer. The activating BRAF p.Val600Glu (p.V600E) variant is commonly seen in sporadic colorectal tumors and rarely reported in LS-related colon cancers (50). The presence of this oncogenic variant in the absence of an LS-related signature serves as evidence against germline variant involvement. Similarly, *TP53* alterations are very common in tumors in humans, with over 91% of tumors exhibiting a second allele loss by single nucleotide variation, chromosomal deletion, or copy neutral-LOH (51). Therefore, the presence of a *TP53* alteration in a tumor of an individual with a germline *TP53* VUS does not serve as stand-alone evidence that the germline variant is the first hit in the gene. In the case of LFS, as presented here, the details of the personal and family history of the patient are critical in the evaluation of *TP53* VUS.

Genetic factors are also essential in this integrated germline somatic evaluation approach. Recent large-scale sequencing obtained from cohorts free from ascertainment bias demonstrated some cancer genes or alleles might not be as highly penetrant as once considered or may exhibit variable expressivity (22, 23, 52–54). These observations suggest the presence of genetic modifiers affecting the presentation of disease, which makes a strong argument in favor of assessing germline variants in parallel with patient’s tumor profile. For example, *MLH1* and *MSH2* genetic variants are reportedly associated with high penetrance LS-related colorectal cancer, whereas *MSH6* and *PMS2* variants are reportedly associated, respectively, with modest or no increased risk of colorectal cancer (54). In the integrated germline and somatic assessment presented here, germline variants in *MSH6* and *PMS2* were assessed with the combination of MMR/MSI/IHC/LOH and personal clinical findings, all of which collectively elucidate a likely involvement of the germline variant in patient cancer. The absence of this strong supporting evidence, as shown in Subject 5-7, was equally valuable for a negative finding. It suggests the germline variants in MMR genes may be polymorphic alterations with no cancer consequence. Alternatively, those germline variants may be consequential but require genetic modifiers, the absence of which in those individuals produces no identifiable cancer consequence.

In conclusion, our approach for germline variant assessment was demonstrably valuable in the clinical management of each patient described herein. To be effective in a wider array of cancer types our approach will require creation of internal and cross-institutional databases, careful collation of detailed somatic and germline genomic data, and the integrated interpretation of germline variants in a larger cohort. The findings would be helpful to assess the classification, penetrance, and variable expressivity of variants in cancer with the goal to help guide clinical management of patients and their families.

## Materials and methods

### Patient cohort

The subjects in this study consisted of patients who presented both at the Division of Cancer Genetics and Prevention, Dana-Farber Cancer Institute (DFCI) for genetic evaluation, and at the Pathology Department, Brigham and Women’s Hospital (BWH) for tumor evaluation between 2016 to 2021. The inclusion criteria included patients with 1) classification of VUS for germline variants and high concern for Lynch-related colon cancer, hereditary paraganglioma-pheochromocytoma syndrome, or Li-Fraumeni syndrome based on the genetic evaluation, and 2) availability of full tumor molecular results and somatic genetic data for these patients. Subjects 1-4 were identified in this cohort. Alternatively, for negative cases the inclusion criteria were: 1) classification of VUS for germline variants in MMR, *SDH*, and *TP53* genes in any individual in the PROACTIVE database regardless of cancer types, and 2) availability of full tumor molecular results and somatic genetic data. Subjects 5-16 were identified in this cohort.

### PROACTIVE database

The PROACTIVE (Profile And Cancer gene Testing for IndiVidual Evaluation) research project is a DFCI institute-wide study aimed at investigating germline genetic data from patients with cancers of diverse types with no association with established hereditary cancer predisposition syndromes. For the study herein, a patient cohort from PROACTIVE included those who had both their germline and tumor DNA evaluated at the Center for Advanced Molecular Diagnostics (CAMD) at BWH. The PROACTIVE database was generated first by extracting separately the germline and somatic OncoPanel variant call data for each patient. Somatic single nucleotide variants (SNV), copy number variants (CNV) and structural variants (SV), and germline SNV and CNV calls were generated through the CAMD variant calling pipelines.

A combined germline and somatic database of variant calls was generated using RStudio by importing previously captured de-identified patient sample files for somatic and germline data. Annotated lists for the somatic and germline data were generated through the data frame command which isolated information of interest. The following categories were selected for isolation of somatic variants: patient coded ID numbers, Oncotree classifier, genes, cDNA changes, protein changes, allelic fraction, coverage, copy number calls, copy number count, left call SV gene, and right call SV gene. Germline variants information was isolated through the following categories: patient coded ID numbers, cytoband location, genes, cDNA changes, protein changes, allelic fraction, and coverage. The merge function was used using the coded patient ID numbers as the base to generate a query database containing all the isolated information from both somatic and germline files. The newly generated master database was used for variant query and analysis.

### Genetic clinical evaluation criteria

Amsterdam II criteria (55) were used for the clinical diagnosis of Lynch syndrome. The criteria were met by the presence of three relatives with any LS-associated cancer, with one being a first-degree relative of the other two, within two successive generations, and at least one diagnosis before age 50.

Bethesda guidelines (56) adapted by NCCN were used to determine if germline evaluation for LS should be performed. The criteria include meeting any of the following: colorectal cancer diagnosed under age 50; the presence of synchronous, metachronous colorectal, or other LS-associated tumors; colorectal cancer with MSI- high histology in a patient diagnosed earlier than age 60; colorectal cancer diagnosed in a patient with at least one first-degree relative with a LS tumor with one of the tumors diagnosed under age 50; or colorectal cancer diagnosed in a patient with at least two or more first/second-degree relatives with LS-associated tumors, regardless of age.

PREMM5 (57) was used to calculate the likelihood of LS germline mutation in any of the following genes: *MLH1, MSH2, MSH6, PMS2*, and *EPCAM.*

The Revised Chompret (2015) criteria (58) by NCCN (59) were used to assess for *TP53* germline testing. Chompret criteria was fulfilled if any of the following criteria was met: patient with LFS tumor (soft tissue sarcoma, osteosarcoma, CNS tumor, breast cancer, adrenocortical carcinoma) before age 46 and having at least one first-degree relative with any of the cancers described above (other than breast) before age 56 or with multiple primaries at any age; patient with LFS multiple tumors (except multiple breast tumors) with first cancer diagnosed before age 46; patient with adrenocortical carcinoma or choroid plexus carcinoma or rhabdomyosarcoma of embryonal anaplastic subtype at any age; patient with breast cancer before age 31.

### Tumor-derived somatic genomic analysis using somatic OncoPanel

Tumor DNA was analyzed using the somatic OncoPanel test (BWH Pathology, MA, USA). OncoPanel is a next-generation sequencing (NGS) test designed for the detection of single-nucleotide variants (SNVs), insertions and deletions (indels), copy number variants (CNVs), and structural variants (SVs) in tumor sample containing at least 20% of tumor nuclei. The sample library was analyzed by massively parallel sequencing using a solution-phase Agilent-SureSelect hybrid capture kit (Agilent, Santa Clara, CA, USA) and an Illumina HiSeq 2500 (Illumina, San Diego, CA, USA) sequencer. Sequence reads were demultiplex by converting raw Illumina output (BCL files) into one bam file per-barcode, per-lane. PICARD is used to produce unaligned bam files with barcode metrics. Individual BAM files were aligned to human genome (hg19) using pair end alignments. Somatic OncoPanel interrogated the exonic sequences of 447 cancer-related genes, and 191 regions across 60 genes for rearrangements. Somatic OncoPanel test is run on tumor biopsies, while it can detect possible germline events due to the presence of normal cells in biopsies, it does not run an integrated algorithm to separate somatic and germline calls. See the section of Analysis of SNV/indel below for details on filtration of possible germline calls. Methods for detection of SNV/indel, CNV, SV, MMR, and MSI by somatic OncoPanel are described below:

#### Analysis of SNV/indel

Somatic SNVs and indels in tumor samples were detected by MuTect and GATK Indelocator (Broad Institute, Cambridge, MA, USA), respectively, as previously described (21, 22). Briefly, annotations of gene, amino acid change, cDNA change were made using the OncoAnnotate tool. The OncoPanel NGS.Rev interface presents all variants after filtering out variants reported in a panel of normal samples or those found in the Exome Sequencing Project (ESP) and/or gnomAD databases with the allele frequency of >0.1% in any sub-population. Variant filtered by those criteria but present in the COSMIC database (COSMIC, Wellcome Sanger, London, UK) at least twice were subsequently rescued. Variant annotation included gene, genome coordinates, reference and alternate alleles, coverage, allele fraction, cDNA, and protein change. An Integrated Genome Viewer (IGV) was used to perform a technical review for all variants. Somatic OncoPanel was validated with a lower limit of detection of 50Xcoverage and 10% variant allele fraction. Variants with low coverage and/or allele fraction calls with less than five reads of support were excluded from the analysis.

#### Analysis of CNV

Somatic CNVs were detected by RobustCNV (DFCI, Boston, MA, USA), as described previously (21, 22). Each baited segment was normalized against the panel of normal, and the Log2 ratios were plotted for visualization in NGS.Rev. Neutral segments were shown with a Log2 ratio on the zero line. The “all chromosome” view was used to show the overall landscape of a sample’s copy number status. Each chromosome was then manually reviewed for chromosome-level, arm-level, and/or focal gains or losses. Appropriate calls were entered with the designation of low amplifications, high amplifications, one copy deletions, or two copy deletions. In general, low amplifications were called at a Log2 ratio ≥ 0.43 and losses at a Log2 ratio ≤ -0.32.

#### Analysis of SV

Somatic chromosomal rearrangements, large indels, and inversions were assessed by BreaKmer (DFCI, Boston, MA, USA) as described previously (21, 22, 60). BreaKmer identifies sequence fragments that do not map to a contiguous region of the reference sequence. SV fragments were presented in NGS.Rev with the gene(s) involved, genome coordinates, the coverages and numbers of reads supporting the variant, and an IGV snapshot for visual confirmation. SV calls with breakpoints that overlapped repetitive regions of the genome were excluded from the analysis. Variants with read support of ≤ 2% (total split and discordant reads/total coverage across breakpoints) were excluded from the analysis. Variants with greater than 2% support were closely reviewed to confirm the variants were unique to the sample (i.e., not identified in unrelated patients or the normal control).

#### Analysis of Mismatch Repair Status (MMR)/Microsatellite Instability (MSI)

MMR status was determined by the number of homopolymer indel counts detected in all reviewed variants. Homopolymer indels are defined as one basepair insertions or deletions that occur adjacent to a homopolymer with a length of three or greater basepairs. The number of homopolymer indels detected in each sample was divided by the size of the exonic target regions in the OncoPanel custom bait set of 1.315 megabases (MB). Samples with a value greater than one were considered MMR-deficient/MSI-high.

### Immunohistochemistry

Immunohistochemistry was performed as per usual clinical staining protocols on formalin-fixed, paraffin-embedded tissue sections cut at a thickness of 4 μm. Following deparaffinization and rehydration, antigen retrieval was performed with citrate buffer in a pressure cooker. Tissues were incubated with primary antibodies followed by secondary detection with commercial kits. The primary antibodies used were as follows: SDHA (1:800, clone 2E3; ab14715, Abcam), SDHB (1:300, clone 21A11; ab14714, Abcam), MLH1 (1:300, clone G1680728; MLH1-L-CE, Leica), PMS2 (1:300, clone MRQ-28; 288m-16, Cell Marque), MSH2 (1:400, clone FE11; NA27, Oncogene), and MSH6 (1:800, clone PU29; NCL-L-MSH6, Leica). The DAKO Envision plus detection system was used for SDHA and SDHB, whereas the Leica Novolink detection system was used for MLH1, PMS2, MSH2, and MSH6.

### Germline genomic analysis

#### Commercial NGS testing

Germline samples for Subjects 1-4 were processed and evaluated by commercial NGS tests:

Subjects 1 and 2 were evaluated by Invitae Hereditary Cancers Panel of 43 genes (CA, USA); Subject 3 was evaluated by panel testing of 12 genes related to paraganglioma risk (*FH, MAX, MEN1, NF1, RET, SHDA, SDHAF2, SDHB, SDHC, SDHD, TMEM127*, and *VHL*) by Invitae (CA, USA) according to published protocols (61). Briefly, samples were enriched for targeted regions using a hybridization-based protocol, and sequenced using the Illumina NovaSeq 6000 platform (Illumina, San Diego, CA, USA Sequence fragments were aligned to the reference human genome (GRCh37). The enrichment was performed on the targeted coding sequences, 10 bp of flanking the intronic sequence (20 bp for BRCA 1/2), and other known causative genomic regions. The exon-level copy number alterations were performed by comparing the read depth for each target sequence with mean read depth and read-depth distribution from a set of clinical samples.

Subject 4 was evaluated by Ambry CancerNext Expanded panel of 67 genes (CA, USA) according to published protocols (62). Briefly, NGS sequencing was performed for all coding regions plus at least five bases into the 5′ and 3′ ends of all the introns and 5′UTR and 3′UTR regions. Sequence enrichment was performed for the target hereditary cancer gene coding exons followed by polymerase chain reaction (RainDance Technologies, Billerica, MA, USA). The enriched libraries were then used for paired-end sequencing using Illumina HiSeq 2000 (Illumina, San Diego, CA). Sequence fragments were aligned to the reference human genome (GRCh37).

#### Germline Oncopanel testing

Germline samples for PROACTIVE data (subjects 5-16) used for this study were processed by germline OncoPanel (BWH Pathology, USA) on DNA obtained peripheral blood. Germline OncoPanel interrogates 147 hereditary cancer genes. Germline sequence BAM files were analyzed by the GATK Haplotype Caller to detect both SNVs and indels, RobustCNV for copy number alterations (with the exon-level calling capability), and BreaKmer to identify germline indels > 5 nt. Similar to the somatic OncoPanel pipeline described above, likely SNPs and artifacts were filtered out of the variant lists by comparing the calls to a panel of 77 normal blood samples (PON) and with the population frequencies in the gnomAD and ESP databases. Any variant found in the PON at 20% and/or in any ethnic sub-population at > 1% that was not defined as pathogenic or likely pathogenic (P/LP) in ClinVar was filtered. Germline samples were manually reviewed in NGS.Rev for visual confirmation of all variants identified by the pipeline tools. The germline Oncopanel is not currently integrated with the somatic Oncopanel pipeline.

## Data availability statement

The original contributions presented in the study are included in the article/**Supplementary Material**, further inquiries can be directed to the corresponding author.

## Ethics statement

The studies involving human participants were reviewed and approved by IRB at Dana Farber Cancer Institute IRB at Mass General Brigham. The patients/participants provided their written informed consent to participate in this study.

## Author contributions

AG, AS, DK, AC, JG contributed to conception and design of the study. AG, CH, MDM, organized the database. AG, AS, DK, AC, JG, DM, RI, CH, FA, MDM, MD, JB, LS, MY, HR performed the analysis and/or clinical evaluation of data AG, AS, DM, DK, AC wrote the first draft of the manuscript. RI wrote sections of the manuscript. All authors contributed to the revision and reading of the manuscript. All authors contributed to the article and approved the submitted version.

## Conflict of interest

The authors declare that the research was conducted in the absence of any commercial or financial relationships that could be construed as a potential conflict of interest.

## Publisher’s note

All claims expressed in this article are solely those of the authors and do not necessarily represent those of their affiliated organizations, or those of the publisher, the editors and the reviewers. Any product that may be evaluated in this article, or claim that may be made by its manufacturer, is not guaranteed or endorsed by the publisher.
