# Supplementary material for: An integrated somatic and germline approach to aid interpretation of germline variants of uncertain significance in cancer susceptibility genes
Source: Front Oncol. 2022 Aug 25;12:942741. doi: 10.3389/fonc.2022.942741 (PMC9453486; doi:10.3389/fonc.2022.942741)
Supplement: Supplementary file 1 [file Table_1.docx]

Supplementary Table 1- Full Clinical History of Study Subjects 1-4

| **Subject 1** | A 71-year-old male presented to genetics in December 2017 due to a recent diagnosis of colon adenocarcinoma. In June 2017, he underwent a colonoscopy and upper endoscopy for iron deficiency anemia. The upper endoscopy exam was negative; however, his colonoscopy identified a mass in the mid ascending colon. Biopsy of this mass revealed adenocarcinoma. DNA mismatch repair testing by immunohistochemistry revealed absent nuclear staining of PMS2 and intact nuclear staining of MLH1, MSH2, and MSH6 proteins. In July 2017, the patient underwent right hemicolectomy. Pathology revealed 8.5cm, Stage IIA (pT3 pN0 cM0), low-grade adenocarcinoma in ascending colon. Somatic OncoPanel was performed on the tumor specimen. This tumor profiling demonstrated an MMR-D/MSI-H tumor, with two variants in the *PMS2* gene, c.716T>G (p.Leu239Arg) with variant allelic fraction (VAF) 49% (later learned to be germline), and *PMS2* c.904-2A>C  with VAF 36%.  In December 2017, he pursued genetic counseling and testing. Germline genetic testing through Invitae’s (CA, USA) Common Hereditary Cancers Panel of 43 genes showed a heterozygous *PMS2* c.716T>G (p.Leu239Arg) that was classified as a variant of uncertain significance by Invitae. The patient’s family history was notable for colon cancer in his brother at age 53, and either colon cancer or stomach cancer diagnosed in his father in his 40s. The paternal family structure is small with limited family health information. The maternal lineage was not suggestive of a hereditary cancer predisposition. Based on the isolated loss of PMS2 protein by immunohistochemistry (IHC), a somatic pathogenic variant in *PMS2* and germline variant in *PMS2* that is concerning for pathogenicity, in the context of a personal and family history of colon cancer, this patient has been managed with presumed Lynch syndrome. |
| --- | --- |
| **Subject 2** | The patient initially presented to Genetics after his diagnosis of a 14 mm polyp in the descending colon at age 44 confirmed by pathology to be a sigmoid colon cancer. Prior colonoscopy at age 38 was normal. IHC of this tumor showed loss of MSH6 and presence of MLH1, MSH2, and PMS2 proteins in the tumor. Surgery requested genetic evaluation prior to surgical intervention. A three-generation pedigree was collected (Figure 1). The patient reported that his father was diagnosed with colon cancer at 42, pancreatic cancer at 66 and died at 66. The patient’s maternal grandmother was diagnosed with breast cancer at 73 and died at 75. He underwent genetic testing through Invitae’s (CA, USA) Common Hereditary Cancers Panel analysis and was found to have a variant of uncertain significance in *MSH6*, c.1439_14441dup (p.Val480dup). Based on these findings and in the context of a personal and family history of colon cancer, this patient has been managed with presumed Lynch syndrome. |
| **Subject 3** | Patient initially presented to the ER at age 43 with chest pain and was diagnosed with a 7.0 cm right atrial paraganglioma. IHC showed intact staining for SDHA and absent staining for SDHB, raising concern for a germline variant in SDHB, SDHC, or SDHD proteins. She was referred for genetic counseling to discuss germline testing options. A three-generation pedigree was collected (Figure 1). She reported that her sibling died in infancy and had a tumor in her stomach, but the tumor type was unknown. Her father was also diagnosed with prostate cancer in his 70s. There was no additional family history of cancer or tumors. The patient reported one sudden death in the family – her maternal uncle who died in his teens due to drowning.    The patient proceeded with germline panel testing of 12 genes related to paraganglioma risk (*FH, MAX, MEN1, NF1, RET, SHDA, SDHAF2, SDHB, SDHC, SDHD, TMEM127,* and *VHL*) at Invitae’s (CA, USA). Results identified a heterozygous *SDHC* c.374T>G (p.Met125Arg) which was classified as a variant of uncertain significance by the reporting laboratory. The somatic testing of her paraganglioma identified a single copy deletion of the *SDHC* gene in addition to the *SDHC* c.374T>G (p.Met125Arg) variant in 67% of 402 reads, indicating loss of heterozygosity of *SDHC* in her tumor. Due to the abnormal IHC results, loss of heterozygosity (LOH) on somatic profiling, and the patient’s personal history of a rare component tumor, the germline *SDHC* variant was treated as a clinically significant finding. Screening for *SDHC*-associated hereditary paraganglioma-pheochromocytoma syndrome was recommended for the patient and cascade testing was recommended for family members. |
| **Subject 4** | A 60-year-old female presented to genetics in July 2020 for updated genetic testing due to her personal history of breast cancer at age 40 and family history of cancer notable for early onset breast cancer, prostate cancer, glioblastoma, and colon cancer. She previously had negative *BRCA*1/2 testing in 2009. The patient elected to proceed with multigene panel testing with Ambry’s (CA, USA) CancerNext Expanded panel of 67 genes. Testing revealed a heterozygous *TP53* c.640C>T (p.His214Tyr) reported as variant of uncertain significance by the laboratory. The patient met Chompret criteria specified by NCCN guidelines with breast cancer diagnosed before age 46 and a first degree relative with a tumor from the Li-Fraumeni (LFS) spectrum before age 56, which includes her brother who died from a glioblastoma diagnosed at age 38. The patient’s sister died with a cancer history that also was suggestive of an LFS phenotype with breast cancer diagnosed at age 45, contralateral breast cancer at age 50, and leukemia at age 60. With permission to view her records, the sister had a somatic OncoPanel performed on malignant cells of her bone marrow. This somatic OncoPanel reported *TP53* c.640C>T (p.His214Tyr) (presumed to be germline as identified in her sister) and a second *TP53* c.814G>A (p.V272M) variant. Additionally, their brother who is living had *TP53* testing done through City of Hope laboratory in 2009 due to his history of prostate cancer diagnosed at age 50. Their brother had updated multigene panel testing with Ambry Genetics CancerNext panel in 2020 that reported the *TP53* variant c.640C>T (p.His214Tyr) as a variant of uncertain significance, no other variants were reported. Since family member testing has been pursued, those who were positive have been followed with LFS management guidelines. The female patient first described in this case underwent esophagogastroduodenoscopy (EGD) and colonoscopy per LFS management guidelines which revealed a stage IIIb adenocarcinoma of the ascending colon. The phenotype of this family presenting with those affected with cancer meeting Chompret criteria in the setting of a *TP53* variant raises concern of pathogenicity of this familial variant. |
