# Supplementary material for: An integrated somatic and germline approach to aid interpretation of germline variants of uncertain significance in cancer susceptibility genes
Source: Front Oncol. 2022 Aug 25;12:942741. doi: 10.3389/fonc.2022.942741 (PMC9453486; doi:10.3389/fonc.2022.942741)
Supplement: Supplementary file 2 [file Table_2.docx]

| **Subjects** | **Germline alteration** | **RefSeq Transcript** | **Classification in ClinVAr** | **Clinvar ID** |
| --- | --- | --- | --- | --- |
| 1 | *PMS2*: c.716T>G (p.Leu239Arg) | NM_000535.7 | Uncertain significance | 411058 |
| 2 | *MSH6*:c.1439_1441dup (p.Val480dup) | NM_000179.3 | Uncertain significance | 1009858 |
| 3 | *SDHC*:c.374T>G (p.Met125Arg) | NM_003001.5 | Uncertain significance | 407057 |
| 4 | *TP53*:c.640C>T (p.His214Tyr) | NM_000546.6 | Uncertain significance | 1053808 |
| 5 | *PMS2*:c.620G>A (p.Gly207Glu) | NM_000535.7 | Conflicting interpretations of pathogenicity  Benign(5);Likely benign(2);Uncertain significance(6) | 127793 |
| 6 | *PMS2*:c.1688_1689delinsAG (p.Arg563Gln) | NM_000535.7 | Conflicting interpretations of pathogenicity  Likely benign(3);Uncertain significance(2) | 135940 |
| 7 | *MSH6*:c.1028C>T (p.Pro343Leu) | NM_000179.3 | Uncertain significance | 127552 |
| 8 | *MSH6*:c.494T>G (p.Phe165Cys) | NM_000179.3 | Uncertain significance | 231130 |
| 9 | *MSH6*:c.1774G>A (p.Val592Ile) | NM_000179.3 | Uncertain significance | 628131 |
| 10 | *MSH6*:c.3256C>T (p.Pro1086Ser) | NM_000179.3 | Uncertain significance | 525751 |
| 11 | *SDHA*:c.1579C>T (p.Arg527Cys) | NM_004168.4 | Uncertain significance | 412340 |
| 12,13 | *SDHC*:c.430G>C(p.Glu144Gln) | NM_001035511.2 | Conflicting interpretations of pathogenicity  Benign(5);Likely benign(3);Uncertain significance(1) | 41773 |
| 14 | *TP53*:c.-29+236T>C  *TP53*:c.-29+571T>C | NM_000546.6 | N/A  N/A | N/A  N/A |
| 15, 16 | *TP53*:c.-29+1044T>A | NM_000546.6 | N/A | N/A |

**Supplementary Table 2: Annotated Germline Variants in Study Subjects**
